# Supplementary material for: The Evolution and Characterization of the RNA Interference Pathways in Lophotrochozoa
Source: Genome Biol Evol. 2024 May 7;16(5):evae098. doi: 10.1093/gbe/evae098 (PMC11114477; doi:10.1093/gbe/evae098)
Supplement: evae098_Supplementary_Data [file evae098_supplementary_data.zip › Supplementary_materials.zip!/selected_miRNAs/NoGe_selected_miRNAs/mirdeep_pdf_files/NMRB01001254.1_Notospermus_geniculatus_strain_Ushimado_scaffold1254,_whole_genome_shotgun_sequence_26702.pdf]

| | | |
|---|---|---|
| Provisional ID | : | NMRB01001254.1_Notospermus_geniculatus_strain_Ushimado_scaffold1254,_whole_genome_shotgun_sequence_26702 |
| Score total | : | 43184.5 |
| Score for star read(s) | : | 3.9 |
| Score for read counts | : | 43176.2 |
| Score for mfe | : | 1.4 |
| Score for randfold | : | 0 |
| Score for cons. seed | : | 3 |
| Total read count | : | 84700 |
| Mature read count | : | 84672 |
| Loop read count | : | 0 |
| Star read count | : | 28 |

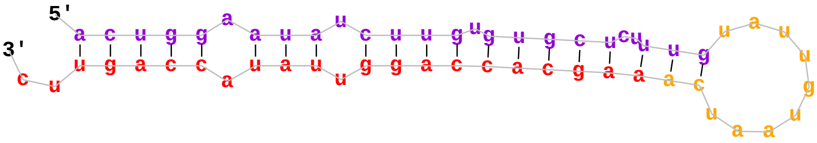


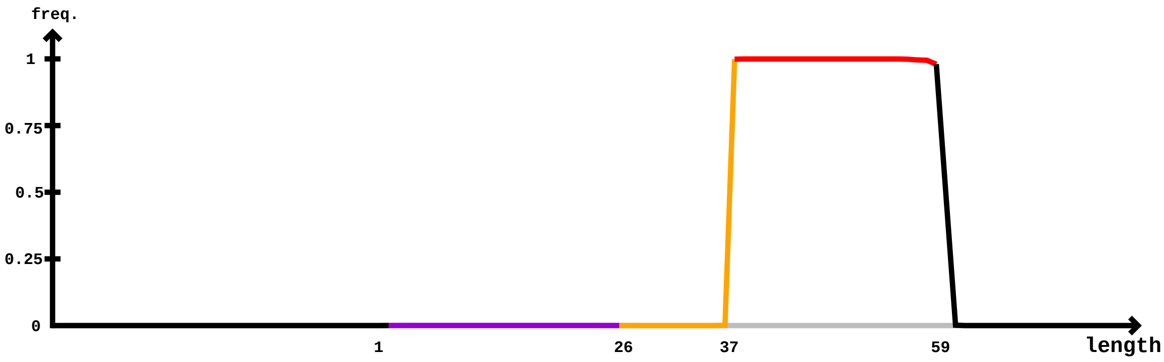


**Star** **Mature**

| | | | |
|---|---|---|---|
| 5'- aaggcuuacccuguccuagcaucaguuuugcgaacuggaauaucuugugugcucuuuguauuguaaucaaagcaccagguuauaccaguucgugaucugguccgugcaaua -3' | obs | | |
| aaggcuuacccuguccuagcaucaguuuugcgaacuggaauaucuugugugcucuuuguauuguaaucaaagcaccagguuauaccaguucgugaucugguccgugcaaua | exp | | |
| .((((.......).))).(((((((..(((((((((((.(((.((((.(((((..(((.........)))))))))))).))).))))))))))).)))....))))....  | reads | mm | sample |
| .................................acuggaauaucuugugugcucuu....................................................... | 1 | 0 | Mm3 |
| .................................acuggaauaucuugugugcucuuug..................................................... | 6 | 0 | Mm3 |
| ..........................................................uauuguaaucaaagcaccagguuauacc......................... | 1 | 0 | Mm3 |
| ....................................................................aaagcaccagguuauaccaguu..................... | 2 | 0 | Mm3 |
| ....................................................................aaagcaccagguuauaccaguuc.................... | 1 | 0 | Mm3 |
| ....................................................................Caagcaccagguuauaccaguuc.................... | 2 | 1 | Mm3 |
| .....................................................................aagcaccagguuauacca........................ | 5 | 0 | Mm3 |
| .....................................................................aagcaccagguuauaccagu...................... | 10 | 0 | Mm3 |
| .....................................................................aagcaccagguuauacAagu...................... | 1 | 1 | Mm3 |
| .....................................................................aagcaccagguuauaccaguu..................... | 104 | 0 | Mm3 |
| .....................................................................aagcaccagguuauaccaAuu..................... | 1 | 1 | Mm3 |
| .....................................................................Uagcaccagguuauaccaguuc.................... | 4 | 1 | Mm3 |
| .....................................................................aagcaccagguuauaccaguuA................... | 3 | 1 | Mm3 |
| .....................................................................aUgcaccagguuauaccaguuc.................... | 3 | 1 | Mm3 |
| .....................................................................aagUaccagguuauaccaguuc.................... | 1 | 1 | Mm3 |
| .....................................................................aagcaccUgguuauaccaguuc.................... | 2 | 1 | Mm3 |
| .....................................................................aagcaccGgguuauaccaguuc.................... | 2 | 1 | Mm3 |
| .....................................................................Gagcaccagguuauaccaguuc.................... | 2 | 1 | Mm3 |
| .....................................................................aagcaccagguuaAaccaguuc.................... | 1 | 1 | Mm3 |
| .....................................................................aagcaccagguuauacUaguuc.................... | 30 | 1 | Mm3 |
| .....................................................................aagcaccagguuauaccagCuc.................... | 1 | 1 | Mm3 |
| .....................................................................aagcacUagguuauaccaguuc.................... | 3 | 1 | Mm3 |
| .....................................................................aagcaccaggAuauaccaguuc.................... | 14 | 1 | Mm3 |
| .....................................................................Nagcaccagguuauaccaguuc.................... | 1 | 1 | Mm3 |
| .....................................................................aagcaccagguuauaccGguuc.................... | 21 | 1 | Mm3 |
| .....................................................................aagcaccagguuauUccaguuc.................... | 2 | 1 | Mm3 |
| .....................................................................aagcaccaggGuauaccaguuc.................... | 6 | 1 | Mm3 |
| .....................................................................aagcaccagguuauGccaguuc.................... | 2 | 1 | Mm3 |
| .....................................................................aagcaccagguuauaccCguuc.................... | 24 | 1 | Mm3 |
| .....................................................................aagcaccagguuauaccaguCc.................... | 1 | 1 | Mm3 |
| .....................................................................aagcaccaggCuauaccaguuc.................... | 7 | 1 | Mm3 |
| .....................................................................aagAaccagguuauaccaguuc.................... | 1 | 1 | Mm3 |
| .....................................................................Cagcaccagguuauaccaguuc.................... | 1 | 1 | Mm3 |

Star Mature

| | | | |
|---|---|---|---|
| aaggcuuacccuguccuagcaucaguuuugcgaacuggaauaucuugugugcucuuuguauuguaaucaaagcaccagguuauaccaguucgugaucugguccgugcaaua | | | |
| .....................................................................aagcaNcagguuauaccaguuc.................... | 2 | 1 | Mm3 |
| .....................................................................aagcaccaUguuauaccaguuc.................... | 1 | 1 | Mm3 |
| .....................................................................aagcaccagguuauacAaguuc.................... | 21 | 1 | Mm3 |
| .....................................................................aagcaccagguuauaccaUuuc.................... | 1 | 1 | Mm3 |
| .....................................................................aagcaccagUuuauaccaguuc.................... | 2 | 1 | Mm3 |
| .....................................................................aaUcaccagguuauaccaguuc.................... | 1 | 1 | Mm3 |
| .....................................................................aagcaUcagguuauaccaguuc.................... | 1 | 1 | Mm3 |
| .....................................................................aagcaccagguuauaccaguuU.................... | 9 | 1 | Mm3 |
| .....................................................................aagcaccagguuauacGaguuc.................... | 13 | 1 | Mm3 |
| .....................................................................aagcaccagguuauaccaguuc.................... | 5641 | 0 | Mm3 |
| .....................................................................aagcaccaAguuauaccaguuc.................... | 1 | 1 | Mm3 |
| .....................................................................aagcaccagguuauaUcaguuc.................... | 1 | 1 | Mm3 |
| .....................................................................aaAcaccagguuauaccaguuc.................... | 1 | 1 | Mm3 |
| .....................................................................aagcaccagguuauaccUguuc.................... | 9 | 1 | Mm3 |
| .....................................................................aagcaccagguuaCaccaguuc.................... | 2 | 1 | Mm3 |
| .....................................................................aagcaccagguuauaccaguucC................... | 4 | 1 | Mm3 |
| .....................................................................aagcaccagguuauaccaguucU................... | 290 | 1 | Mm3 |
| .....................................................................aagcaccagguuauaccaguucCu.................. | 1 | 1 | Mm3 |
| .....................................................................aagcaccagguuauaccaguucUu.................. | 7 | 1 | Mm3 |
| ......................................................................agcaccagguuauaccaguuc.................... | 7 | 0 | Mm3 |
| .......................................................................gcaccagguuauaccaguuc.................... | 1 | 0 | Mm3 |
| ............Auccuagcaucaguuuugcga.............................................................................. | 3 | 1 | Fm3 |
| .................................acuggaauaucuugugugcucuu....................................................... | 1 | 0 | Fm3 |
| ....................................................................Caagcaccagguuauaccaguuc.................... | 1 | 1 | Fm3 |
| .....................................................................aagcaccagguuauacca........................ | 19 | 0 | Fm3 |
| .....................................................................aagcaccagguuauaccag....................... | 195 | 0 | Fm3 |
| .....................................................................aGgcaccagguuauaccag....................... | 1 | 1 | Fm3 |
| .....................................................................aagcaccGgguuauaccag....................... | 1 | 1 | Fm3 |
| .....................................................................aagcaccaUguuauaccag....................... | 1 | 1 | Fm3 |
| .....................................................................aagcaccagguuauaccCg....................... | 2 | 1 | Fm3 |
| .....................................................................aagcaccagguuauaccaU....................... | 5 | 1 | Fm3 |
| .....................................................................aagcaccagguuauacAag....................... | 2 | 1 | Fm3 |
| .....................................................................aagcaccagguuauaUcagu...................... | 3 | 1 | Fm3 |
| .....................................................................Uagcaccagguuauaccagu...................... | 1 | 1 | Fm3 |
| .....................................................................aagcaccagguuauaccagC...................... | 1 | 1 | Fm3 |
| .....................................................................aagcaccagguuauaccUgu...................... | 1 | 1 | Fm3 |
| .....................................................................aagcaccagguuauaccagu...................... | 27 | 0 | Fm3 |
| .....................................................................aagcaccagguuauaccGguu..................... | 2 | 1 | Fm3 |
| .....................................................................aagcaccagguuauacAaguu..................... | 2 | 1 | Fm3 |
| .....................................................................aagcaccagguuauaccaguu..................... | 334 | 0 | Fm3 |
| .....................................................................aagcaccagguuauGccaguu..................... | 1 | 1 | Fm3 |
| .....................................................................aagcaccaggAuauaccaguu..................... | 2 | 1 | Fm3 |
| .....................................................................aagcaccagguuauaccUguu..................... | 2 | 1 | Fm3 |
| .....................................................................aagAaccagguuauaccaguu..................... | 1 | 1 | Fm3 |
| .....................................................................aagcaccagguuauacUaguu..................... | 2 | 1 | Fm3 |
| .....................................................................aagcaAcagguuauaccaguu..................... | 1 | 1 | Fm3 |
| .....................................................................aagcaccaggGuauaccaguu..................... | 1 | 1 | Fm3 |
| .....................................................................Gagcaccagguuauaccaguu..................... | 1 | 1 | Fm3 |
| .....................................................................aagcaccagguuauacGaguu..................... | 1 | 1 | Fm3 |
| .....................................................................aagcaccagguGauaccaguu..................... | 2 | 1 | Fm3 |
| .....................................................................aagcaccagguuauaccaguuc.................... | 2489 | 0 | Fm3 |
| .....................................................................aagcaccagguCauaccaguuc.................... | 2 | 1 | Fm3 |
| .....................................................................aagcaccagguuauacAaguuc.................... | 12 | 1 | Fm3 |
| .....................................................................aaAcaccagguuauaccaguuc.................... | 1 | 1 | Fm3 |
| .....................................................................aCgcaccagguuauaccaguuc.................... | 1 | 1 | Fm3 |
| .....................................................................aagcaccagguuauaccagAuc.................... | 2 | 1 | Fm3 |
| .....................................................................aagcacAagguuauaccaguuc.................... | 2 | 1 | Fm3 |
| .....................................................................aagcaccaggCuauaccaguuc.................... | 2 | 1 | Fm3 |
| .....................................................................aagcaccagguuauaccGguuc.................... | 10 | 1 | Fm3 |
| .....................................................................aagcaccagguuauaccaguuU.................... | 5 | 1 | Fm3 |
| .....................................................................aagcaccagguuauaccaguuG.................... | 1 | 1 | Fm3 |
| .....................................................................aagcaccagguuauaccaguAc.................... | 1 | 1 | Fm3 |
| .....................................................................aagcaccagguuauacUaguuc.................... | 10 | 1 | Fm3 |
| .....................................................................aagAaccagguuauaccaguuc.................... | 2 | 1 | Fm3 |
| .....................................................................aaCcaccagguuauaccaguuc.................... | 1 | 1 | Fm3 |
| .....................................................................aagcaccagguuGuaccaguuc.................... | 1 | 1 | Fm3 |
| .....................................................................aagcaccaggAuauaccaguuc.................... | 9 | 1 | Fm3 |
| .....................................................................aagGaccagguuauaccaguuc.................... | 2 | 1 | Fm3 |
| .....................................................................aagcaccagguuauaccUguuc.................... | 12 | 1 | Fm3 |

```
                                 Star                               Mature
aaggcuuacccuguccuagcaucaguuuugcgaacuggaauaucuugugugcucuuuguauuguaaucaaagcaccagguuauaccaguucgugaucugguccgugcaaua
.....................................................................aagcaccagguuauaccCguuc....................    4    1    Fm3
.....................................................................aagcaccagguuaAaccaguuc....................    1    1    Fm3
.....................................................................aagcaccaggGuauaccaguuc....................    3    1    Fm3
.....................................................................aagcaccagguuauacGaguuc....................    2    1    Fm3
.....................................................................aagcaccagguuauaccaguGc....................    1    1    Fm3
.....................................................................aagcaccagguuaCaccaguuc....................    1    1    Fm3
.....................................................................Gagcaccagguuauaccaguuc....................    1    1    Fm3
.....................................................................aagcaccagguuauaUcaguuc....................    1    1    Fm3
.....................................................................aagUaccagguuauaccaguuc....................    1    1    Fm3
.....................................................................aaUcaccagguuauaccaguuc....................    1    1    Fm3
.....................................................................aagcaccGgguuauaccaguuc....................    1    1    Fm3
.....................................................................aagcaccagguuauaccaguucU...................    74   1    Fm3
.....................................................................aagcaccagguuauaccaguucC...................    3    1    Fm3
.....................................................................aagcaccagguuauaccaguucUu..................    1    1    Fm3
......................................................................agcaccagguuauaccaguuc....................    1    0    Fm3

.................................acuggaauaucuugugugcucuu.......................................................    2    0    Mm2
.................................acuggaauaucuugugugcucuuu......................................................    4    0    Mm2
.................................acuggaauaucuugugugcucuuug.....................................................    6    0    Mm2
..........................................................uauuguaaucaaagcaccagguu..............................    2    0    Mm2
....................................................................aaagcaccagguuauaccaguuc....................    13   0    Mm2
....................................................................CaagcaccagguuauaccaguucC...................    23   1    Mm2
.....................................................................aagcaccagguuauacca........................    11   0    Mm2
.....................................................................aagcaccagguuauaccUg.......................    1    1    Mm2
.....................................................................aagcaccagguuauaccaU.......................    1    1    Mm2
.....................................................................aagcaccagguuauaccag.......................    10   0    Mm2
.....................................................................aagcaccagguuauaccagu......................    36   0    Mm2
.....................................................................aagcaccagguuauaccUgu......................    1    1    Mm2
.....................................................................aagcaccagAuuauaccaguu.....................    1    1    Mm2
.....................................................................aagcaccaggAuauaccaguu.....................    1    1    Mm2
.....................................................................aagcaccagguuauaccGguu.....................    1    1    Mm2
.....................................................................aagcaccagguuauaccUguu.....................    1    1    Mm2
.....................................................................aagcaccaggGuauaccaguu.....................    1    1    Mm2
.....................................................................aagcaccagguuauaccCguu.....................    2    1    Mm2
.....................................................................aagcaccagguuauaccaguu.....................    346  0    Mm2
.....................................................................aagcaccagguuauacAaguu.....................    4    1    Mm2
.....................................................................aagcaccagguuauacUaguu.....................    1    1    Mm2
.....................................................................aagcaccagguuauacGaguuc....................    53   1    Mm2
.....................................................................aagcaccUgguuauaccaguuc....................    5    1    Mm2
.....................................................................Uagcaccagguuauaccaguuc....................    21   1    Mm2
.....................................................................aagcaccagguuGuaccaguuc....................    4    1    Mm2
.....................................................................aagcaccagAuuauaccaguuc....................    11   1    Mm2
.....................................................................aagcaccagguuaCaccaguuc....................    8    1    Mm2
.....................................................................aagcaccagguuauaccagAuc....................    5    1    Mm2
.....................................................................aagcaccCgguuauaccaguuc....................    1    1    Mm2
.....................................................................aagcaccagguuauaccaguCc....................    5    1    Mm2
.....................................................................aaUcaccagguuauaccaguuc....................    9    1    Mm2
.....................................................................aagGaccagguuauaccaguuc....................    2    1    Mm2
.....................................................................aagcaccagguuauaccaguuU....................    22   1    Mm2
.....................................................................aagcaNcagguuauaccaguuc....................    5    1    Mm2
.....................................................................aagcaccagguuauUccaguuc....................    7    1    Mm2
.....................................................................aagcaccagguGauaccaguuc....................    3    1    Mm2
.....................................................................aaCcaccagguuauaccaguuc....................    4    1    Mm2
.....................................................................aagAaccagguuauaccaguuc....................    10   1    Mm2
.....................................................................aUgcaccagguuauaccaguuc....................    9    1    Mm2
.....................................................................Nagcaccagguuauaccaguuc....................    21   1    Mm2
.....................................................................aagcaccagguuaAaccaguuc....................    8    1    Mm2
.....................................................................aagUaccagguuauaccaguuc....................    6    1    Mm2
.....................................................................aagcaccagguuCuaccaguuc....................    2    1    Mm2
.....................................................................aCgcaccagguuauaccaguuc....................    2    1    Mm2
.....................................................................aagcGccagguuauaccaguuc....................    4    1    Mm2
.....................................................................aagcaccagguuauaccagCuc....................    3    1    Mm2
.....................................................................aagcacNagguuauaccaguuc....................    2    1    Mm2
.....................................................................aagcaccaggCuauaccaguuc....................    39   1    Mm2
.....................................................................Gagcaccagguuauaccaguuc....................    8    1    Mm2
.....................................................................aagcaccaggAuauaccaguuc....................    77   1    Mm2
.....................................................................aagcaccagCuuauaccaguuc....................    1    1    Mm2
.....................................................................aagcaccagguuauGccaguuc....................    4    1    Mm2
.....................................................................aaAcaccagguuauaccaguuc....................    9    1    Mm2
.....................................................................aagcaccagguuauaccaguuA....................    8    1    Mm2
```

```
                                Star                              Mature
aaggcuuacccuguccuagcaucaguuuugcgaacuggaauaucuugugugcucuuuguauuguaaucaaagcaccagguuauaccaguucgugaucugguccgugcaaua
.....................................................................aagcaccagguuauaccaguAc....................   1       1    Mm2
.....................................................................aagcaccagUuuauaccaguuc....................   4       1    Mm2
.....................................................................aagcaccagguuaGaccaguuc....................   1       1    Mm2
.....................................................................aagcaccagguuauaUcaguuc....................   9       1    Mm2
.....................................................................aagcaccagguuauaccCguuc....................   134     1    Mm2
.....................................................................aagcaGcagguuauaccaguuc....................   1       1    Mm2
.....................................................................aagcaccagguuauaccaguuc....................   39840   0    Mm2
.....................................................................aagcaUcagguuauaccaguuc....................   9       1    Mm2
.....................................................................aagcaccagguAauaccaguuc....................   3       1    Mm2
.....................................................................aagcaccagguuauaccaCuuc....................   1       1    Mm2
.....................................................................aagcaccGgguuauaccaguuc....................   23      1    Mm2
.....................................................................aagcaccagguuauaccaAuuc....................   3       1    Mm2
.....................................................................aagcaccaCguuauaccaguuc....................   1       1    Mm2
.....................................................................aagcaccaUguuauaccaguuc....................   4       1    Mm2
.....................................................................aagcaccagguuauaccaUuuc....................   5       1    Mm2
.....................................................................aagcaccagguuUuaccaguuc....................   6       1    Mm2
.....................................................................aagcaccaggGuauaccaguuc....................   50      1    Mm2
.....................................................................aagcUccagguuauaccaguuc....................   1       1    Mm2
.....................................................................aagcacAagguuauaccaguuc....................   1       1    Mm2
.....................................................................aGgcaccagguuauaccaguuc....................   11      1    Mm2
.....................................................................aagcaccagguuauaccUguuc....................   112     1    Mm2
.....................................................................aagcaccagguuauacAaguuc....................   174     1    Mm2
.....................................................................aagcaccagguCauaccaguuc....................   1       1    Mm2
.....................................................................aagcCccagguuauaccaguuc....................   3       1    Mm2
.....................................................................Cagcaccagguuauaccaguuc....................   3       1    Mm2
.....................................................................aagcacUagguuauaccaguuc....................   13      1    Mm2
.....................................................................aagcaccagguuauacUaguuc....................   101     1    Mm2
.....................................................................aagcaccagguuauaccGguuc....................   111     1    Mm2
.....................................................................aagcaccagguuauaccaguucU...................   1552    1    Mm2
.....................................................................aagcaccagguuauaccaguucC...................   67      1    Mm2
.....................................................................aagcaccagguuauaccaguucg...................   15      0    Mm2
.....................................................................aagcaccagguuauaccaguucUu..................   38      1    Mm2
......................................................................agcaccagguuauaccagu......................   1       0    Mm2
......................................................................agcaccagguuauaccaguuc....................   26      0    Mm2
......................................................................agcacUagguuauaccaguuc....................   1       1    Mm2
......................................................................aAcaccagguuauaccaguuc....................   1       1    Mm2
......................................................................agcaccagguuauaccaguucU...................   2       1    Mm2
.......................................................................gcaccagguuauaccagu......................   1       0    Mm2
........................................................................caccagguuauaccaguuc....................   1       0    Mm2

.....................................................................aagcaccagguuauacca........................   19      0    Mm1
.....................................................................aagcaccagguuauaccag.......................   5       0    Mm1
.....................................................................aagcaccagguuauaccCg.......................   1       1    Mm1
.....................................................................aagcaccagguuauaccagu......................   6       0    Mm1
.....................................................................aagcaccagguuauaccCguu.....................   1       1    Mm1
.....................................................................aagcaccaggGuauaccaguu.....................   1       1    Mm1
.....................................................................aagcaccagguuauaccaguu.....................   255     0    Mm1
.....................................................................aagcaccagguuauacAaguu.....................   2       1    Mm1
.....................................................................aagcaccagguuauaccUguu.....................   1       1    Mm1
.....................................................................aagcacUagguuauaccaguu.....................   1       1    Mm1
.....................................................................aagcaccagguuauaccGguu.....................   1       1    Mm1
.....................................................................aagcaccagguuauacGaguu.....................   1       1    Mm1
.....................................................................aagcaccagguuauacUaguu.....................   1       1    Mm1
.....................................................................aagcaccagguuauaccaguuU....................   3       1    Mm1
.....................................................................aagcaccaggAuauaccaguuc....................   13      1    Mm1
.....................................................................aagAaccagguuauaccaguuc....................   1       1    Mm1
.....................................................................aagcaccagguuUuaccaguuc....................   1       1    Mm1
.....................................................................aagcaccagguuGuaccaguuc....................   1       1    Mm1
.....................................................................aagcaccagguuauUccaguuc....................   3       1    Mm1
.....................................................................aagcaccUgguuauaccaguuc....................   1       1    Mm1
.....................................................................aGgcaccagguuauaccaguuc....................   1       1    Mm1
.....................................................................aagcGccagguuauaccaguuc....................   1       1    Mm1
.....................................................................aaAcaccagguuauaccaguuc....................   3       1    Mm1
.....................................................................aagcaccagguuauaccCguuc....................   27      1    Mm1
.....................................................................aagcaccaAguuauaccaguuc....................   1       1    Mm1
.....................................................................aagcaccagguuauacGaguuc....................   11      1    Mm1
.....................................................................aagcaNcagguuauaccaguuc....................   1       1    Mm1
.....................................................................aagcacUagguuauaccaguuc....................   2       1    Mm1
.....................................................................aagcaccagguuauaccaguuc....................   6477    0    Mm1
.....................................................................aUgcaccagguuauaccaguuc....................   1       1    Mm1
```

| | Star | | Mature | | | | |
|---|---|---|---|---|---|---|---|

| | | | |
|---|---|---|---|
| aaggcuuacccuguccuagcaucaguuuugcgaacuggaauaucuugugugcucuuuguauuguaaucaaagcaccagguuauaccaguucgugaucugguccgugcaaua | | | |
| .....................................................................aagcaccagguuauaccUguuc.................... | 21 | 1 | Mm1 |
| .....................................................................aaUcaccagguuauaccaguuc.................... | 2 | 1 | Mm1 |
| .....................................................................aagcaccagguCauaccaguuc.................... | 2 | 1 | Mm1 |
| .....................................................................aagcaccaggCuauaccaguuc.................... | 6 | 1 | Mm1 |
| .....................................................................Nagcaccagguuauaccaguuc.................... | 5 | 1 | Mm1 |
| .....................................................................aagcaccagAuuauaccaguuc.................... | 2 | 1 | Mm1 |
| .....................................................................aagcacNagguuauaccaguuc.................... | 1 | 1 | Mm1 |
| .....................................................................aagcaccagguuaAaccaguuc.................... | 4 | 1 | Mm1 |
| .....................................................................Uagcaccagguuauaccaguuc.................... | 4 | 1 | Mm1 |
| .....................................................................aagcaccagguuauaccaUuuc.................... | 1 | 1 | Mm1 |
| .....................................................................aagcaccagguuauaccGguuc.................... | 27 | 1 | Mm1 |
| .....................................................................aCgcaccagguuauaccaguuc.................... | 1 | 1 | Mm1 |
| .....................................................................aagcaccagguuauCccaguuc.................... | 1 | 1 | Mm1 |
| .....................................................................aagcaccagguuauacAaguuc.................... | 24 | 1 | Mm1 |
| .....................................................................aagcaccagguuauacUaguuc.................... | 14 | 1 | Mm1 |
| .....................................................................aagcaccagguuauGccaguuc.................... | 1 | 1 | Mm1 |
| .....................................................................aagcaccagguuauaccaguCc.................... | 1 | 1 | Mm1 |
| .....................................................................aagcaccaggGuauaccaguuc.................... | 5 | 1 | Mm1 |
| .....................................................................aagcaccagguuauaccaguucC................... | 36 | 1 | Mm1 |
| .....................................................................aagcaccagguuauaccaguucg................... | 2 | 0 | Mm1 |
| .....................................................................aagcaccagguuauaccaguucU................... | 249 | 1 | Mm1 |
| ......................................................................agcaccagguuauaccaguuc.................... | 1 | 0 | Mm1 |
| ......................................................................agcaccagguuauaccaguucU................... | 3 | 1 | Mm1 |
| .................................acuggaauUucuugugugcucuuug..................................................... | 1 | 1 | Fm1 |
| ....................................................................Caagcaccagguuauaccaguuc.................... | 6 | 1 | Fm1 |
| ....................................................................aaagcaccagguuauaccaguuc.................... | 6 | 0 | Fm1 |
| .....................................................................aagcaccagguuauacca........................ | 13 | 0 | Fm1 |
| .....................................................................aagcaccagguuauaccag....................... | 2 | 0 | Fm1 |
| .....................................................................aagcaccagguuauaccagu...................... | 13 | 0 | Fm1 |
| .....................................................................aagcaccagguuauaccaguu..................... | 92 | 0 | Fm1 |
| .....................................................................aagcaccagguuauaccUguu..................... | 1 | 1 | Fm1 |
| .....................................................................aagcaccagguuauaccGguu..................... | 1 | 1 | Fm1 |
| .....................................................................aagcaNcagguuauaccaguu..................... | 1 | 1 | Fm1 |
| .....................................................................aagcaccagguuauacAaguu..................... | 2 | 1 | Fm1 |
| .....................................................................aagcaccagguuGuaccaguuc.................... | 1 | 1 | Fm1 |
| .....................................................................aaCcaccagguuauaccaguuc.................... | 6 | 1 | Fm1 |
| .....................................................................aagcaccagguuauaUcaguuc.................... | 4 | 1 | Fm1 |
| .....................................................................aCgcaccagguuauaccaguuc.................... | 1 | 1 | Fm1 |
| .....................................................................aUgcaccagguuauaccaguuc.................... | 3 | 1 | Fm1 |
| .....................................................................Uagcaccagguuauaccaguuc.................... | 9 | 1 | Fm1 |
| .....................................................................aagcaccagguuauaccaguAc.................... | 1 | 1 | Fm1 |
| .....................................................................aagcaccaCguuauaccaguuc.................... | 1 | 1 | Fm1 |
| .....................................................................aagcaccaUguuauaccaguuc.................... | 2 | 1 | Fm1 |
| .....................................................................aagcaccagguuauaccaguuU.................... | 12 | 1 | Fm1 |
| .....................................................................aagcaccGgguuauaccaguuc.................... | 17 | 1 | Fm1 |
| .....................................................................aagcUccagguuauaccaguuc.................... | 2 | 1 | Fm1 |
| .....................................................................aagcaccagguuauaccCguuc.................... | 33 | 1 | Fm1 |
| .....................................................................aagcaccagguuUuaccaguuc.................... | 1 | 1 | Fm1 |
| .....................................................................aGgcaccagguuauaccaguuc.................... | 2 | 1 | Fm1 |
| .....................................................................aagcaccagguuauaccaguuc.................... | 13386 | 0 | Fm1 |
| .....................................................................Nagcaccagguuauaccaguuc.................... | 5 | 1 | Fm1 |
| .....................................................................Gagcaccagguuauaccaguuc.................... | 5 | 1 | Fm1 |
| .....................................................................aagcaccagguGauaccaguuc.................... | 2 | 1 | Fm1 |
| .....................................................................aagcaccagguuaGaccaguuc.................... | 1 | 1 | Fm1 |
| .....................................................................aagcGccagguuauaccaguuc.................... | 3 | 1 | Fm1 |
| .....................................................................aagcacUagguuauaccaguuc.................... | 5 | 1 | Fm1 |
| .....................................................................aagcaNcagguuauaccaguuc.................... | 4 | 1 | Fm1 |
| .....................................................................aagcacAagguuauaccaguuc.................... | 3 | 1 | Fm1 |
| .....................................................................aagUaccagguuauaccaguuc.................... | 3 | 1 | Fm1 |
| .....................................................................aaUcaccagguuauaccaguuc.................... | 3 | 1 | Fm1 |
| .....................................................................aagcaccagguuauacAaguuc.................... | 62 | 1 | Fm1 |
| .....................................................................aagcaccUgguuauaccaguuc.................... | 1 | 1 | Fm1 |
| .....................................................................aagcaccagguuauacGaguuc.................... | 25 | 1 | Fm1 |
| .....................................................................aagcaccagguuauacUaguuc.................... | 32 | 1 | Fm1 |
| .....................................................................aagcaccagguuauGccaguuc.................... | 6 | 1 | Fm1 |
| .....................................................................aaAcaccagguuauaccaguuc.................... | 3 | 1 | Fm1 |
| .....................................................................aagcaUcagguuauaccaguuc.................... | 4 | 1 | Fm1 |
| .....................................................................aagcCccagguuauaccaguuc.................... | 2 | 1 | Fm1 |
| .....................................................................aagcaccagguuauaccaguuA.................... | 10 | 1 | Fm1 |

```
                                 Star                                   Mature
aaggcuuacccuguccuagcaucaguuuugcgaacuggaauaucuugugugcucuuuguauuguaaucaaagcaccagguuauaccaguucgugaucugguccgugcaaua
.....................................................................aagcaccagUuuauaccaguuc....................    1      1      Fm1
.....................................................................aagGaccagguuauaccaguuc....................    1      1      Fm1
.....................................................................aagcaccagguuauUccaguuc....................    1      1      Fm1
.....................................................................aagcaccagguuauaccUguuc....................    37     1      Fm1
.....................................................................aagcaccagguuauaccaUuuc....................    3      1      Fm1
.....................................................................aagcaccaggCuauaccaguuc....................    16     1      Fm1
.....................................................................aagcaccaggAuauaccaguuc....................    31     1      Fm1
.....................................................................aagAaccagguuauaccaguuc....................    8      1      Fm1
.....................................................................aagcaccaggGuauaccaguuc....................    19     1      Fm1
.....................................................................aagcaccagguuauaccGguuc....................    54     1      Fm1
.....................................................................aagcaccagguuauaccaguGc....................    1      1      Fm1
.....................................................................aagcaccagguCauaccaguuc....................    2      1      Fm1
.....................................................................aagcaccagguuauaccaguucU...................    745    1      Fm1
.....................................................................aagcaccagguuauaccaguucC...................    85     1      Fm1
.....................................................................aagcaccagguuauaccUguucg...................    1      1      Fm1
.....................................................................aagcaccagguuauaccaguucg...................    9      0      Fm1
.....................................................................aagcaccagguuauaccaguucUu..................    49     1      Fm1
.....................................................................aagcaccagguuauaccaguucCu..................    5      1      Fm1
......................................................................agcaccagguuauaccaguuc....................    2      0      Fm1

.................................acuggaauaucuugugugcucu........................................................    6      0      Fm2
.................................acuggaauaucuugugugcucuuug.....................................................    1      0      Fm2
....................................................................aaagcaccagguuauaccaguuc....................    8      0      Fm2
....................................................................Caagcaccagguuauaccaguuc....................    2      1      Fm2
.....................................................................aagcaccagguuauaccagu......................    4      0      Fm2
.....................................................................aagcaccagguuauaccaUu......................    2      1      Fm2
.....................................................................aagcaccagguuaAaccaguu.....................    1      1      Fm2
.....................................................................aagcaccagguuauaccCguu.....................    1      1      Fm2
.....................................................................aagcaccagguuauaccaguu.....................    78     0      Fm2
.....................................................................aagcaccagguuauGccaguuc....................    1      1      Fm2
.....................................................................aUgcaccagguuauaccaguuc....................    1      1      Fm2
.....................................................................aGgcaccagguuauaccaguuc....................    2      1      Fm2
.....................................................................aagcaccagguuauaccaguGc....................    1      1      Fm2
.....................................................................aagcaccGgguuauaccaguuc....................    6      1      Fm2
.....................................................................aagcaccagguuauaccaUuuc....................    1      1      Fm2
.....................................................................aagcacAagguuauaccaguuc....................    1      1      Fm2
.....................................................................aagcaNcagguuauaccaguuc....................    1      1      Fm2
.....................................................................aagcaccagguuUuaccaguuc....................    1      1      Fm2
.....................................................................aagcaccagguuauUccaguuc....................    1      1      Fm2
.....................................................................aagcacNagguuauaccaguuc....................    1      1      Fm2
.....................................................................aagcaccagguuGuaccaguuc....................    1      1      Fm2
.....................................................................aagcaccagguuauaccaguuG....................    1      1      Fm2
.....................................................................Nagcaccagguuauaccaguuc....................    6      1      Fm2
.....................................................................aagcaccaCguuauaccaguuc....................    1      1      Fm2
.....................................................................aagcaccCgguuauaccaguuc....................    1      1      Fm2
.....................................................................aagcaccagguuauaccagAuc....................    2      1      Fm2
.....................................................................aagcaccaggCuauaccaguuc....................    16     1      Fm2
.....................................................................aagAaccagguuauaccaguuc....................    1      1      Fm2
.....................................................................aagcaccagUuuauaccaguuc....................    1      1      Fm2
.....................................................................aagcaccaggAuauaccaguuc....................    19     1      Fm2
.....................................................................aagcaccagguuauacGaguuc....................    9      1      Fm2
.....................................................................aagcaccagguuauaccaguuA....................    11     1      Fm2
.....................................................................aagcacUagguuauaccaguuc....................    2      1      Fm2
.....................................................................aagcaccagguuauacUaguuc....................    28     1      Fm2
.....................................................................aagcCccagguuauaccaguuc....................    1      1      Fm2
.....................................................................aagcaccagguuaAaccaguuc....................    1      1      Fm2
.....................................................................aagcaccagguuauaUcaguuc....................    1      1      Fm2
.....................................................................Uagcaccagguuauaccaguuc....................    5      1      Fm2
.....................................................................aagcaccaggGuauaccaguuc....................    6      1      Fm2
.....................................................................aagcaccagguuauaccGguuc....................    34     1      Fm2
.....................................................................aagcaccagguGauaccaguuc....................    1      1      Fm2
.....................................................................aagcaccagguuauaccagCuc....................    2      1      Fm2
.....................................................................aagcGccagguuauaccaguuc....................    1      1      Fm2
.....................................................................aagcaccagguuauaccCguuc....................    30     1      Fm2
.....................................................................aagcaUcagguuauaccaguuc....................    1      1      Fm2
.....................................................................aagcaccagguuauaccUguuc....................    22     1      Fm2
.....................................................................aagcaccagguuauaccaguuc....................    8938   0      Fm2
.....................................................................aagcaccagguuauacAaguuc....................    31     1      Fm2
.....................................................................aagcaccagguuaCaccaguuc....................    2      1      Fm2
.....................................................................Gagcaccagguuauaccaguuc....................    2      1      Fm2
```

```
                                 Star                               Mature
aaggcuuacccuguccuagcaucaguuuugcgaacuggaauaucuugugugcucuuuguauuguaaucaaagcaccagguuauaccaguucgugaucugguccgugcaaua
.....................................................................aagcaccagguuauaccaguucC...................   35    1    Fm2
.....................................................................aagcaccagguuauaccaguucU...................   491   1    Fm2
.....................................................................Nagcaccagguuauaccaguucg...................   1     1    Fm2
.....................................................................aagcaccagguuauaccaguucUu..................   11    1    Fm2
......................................................................agcaccagguuauaccaguuc....................   3     0    Fm2
......................................................................agcaccagguuauaccaguucU...................   5     1    Fm2
........................................................................caccagguuauaccaguuc....................   3     0    Fm2
```
